# Radiofrequency assisted pancreaticoduodenectomy for palliative surgical resection of locally advanced pancreatic adenocarcinoma

**DOI:** 10.18632/oncotarget.24596

**Published:** 2018-02-28

**Authors:** Jayant Kumar, Isabella Reccia, Mikael H. Sodergren, Tomokazu Kusano, Artur Zanellato, Madhava Pai, Duncan Spalding, Dimitris Zacharoulis, Nagy Habib

**Affiliations:** ^1^ Department of Surgery and Cancer, Hammersmith Campus, Imperial College London, London, UK; ^2^ Department of General Surgery, University Hospital of Larissa, Larissa, Greece

**Keywords:** pancreatic ductal adenocarcinoma, radiofrequency ablation, pancreaticoduodenectomy, palliation

## Abstract

**Background:**

Despite careful patient selection and preoperative investigations curative resection rate (R0) in pancreaticoduodenectomy ranges from 15% to 87%. Here we describe a new palliative approach for pancreaticoduodenectomy using a radiofrequency energy device to ablate tumor *in situ* in patients undergoing R1/R2 resections for locally advanced pancreatic ductal adenocarcinoma where vascular reconstruction was not feasible.

**Results:**

There was neither postoperative mortality nor significant morbidity. Each time the ablation lasted less than 15 minutes. Following radiofrequency ablation it was observed that the tumor remnant attached to the vessel had shrunk significantly. In four patients this allowed easier separation and dissection of the ablated tumor from the adherent vessel leading to R1 resection. In the other two patients, the ablated tumor did not separate from vessel due to true tumor invasion and patients had an R2 resection. The ablated remnant part of the tumor was left *in situ.*

**Conclusion:**

Whenever pancreaticoduodenectomy with R0 resection cannot be achieved, this new palliative procedure could be considered in order to facilitate resection and enable maximum destruction in remnant tumors.

**Method:**

Six patients with suspected tumor infiltration and where vascular reconstruction was not warranted underwent radiofrequency-assisted pancreaticoduodenectomy for locally advanced pancreatic ductal adenocarcinoma. Radiofrequency was applied across the tumor vertically 5–10 mm from the edge of the mesenteric and portal veins. Following ablation, the duodenum and the head of pancreas were removed after knife excision along the ablated line. The remaining ablated tissue was left *in situ* attached to the vessel.

## INTRODUCTION

Pancreatic ductal adenocarcinoma (PDAC) is the fourth leading cause of cancer deaths in the Western World and accounts for almost 3% of all cancer deaths in the United States [1]. Based on data from surveillance, epidemiology, and end results, the overall 5 years survival is 7.7% [1]. The 5-year survival rate for locally advanced and metastatic pancreatic cancer has been reported as 11.1% and 2.6%, respectively [1]. As surgery is the only curative option for PDAC, and tumor invasion of surrounding vessels, especially superior mesenteric vein (SMV) and portal vein (PV) is common, pancreaticoduodenectomy (PD) with vascular resection and reconstruction for locally advanced PDAC has been advocated to improve results [2].

One of the foremost prognostic factors affecting long-term survival in patients with PDAC is to achieve negative resection margins (R0 resection) [3, 4]. Thus, surgery with curative intent should be recommended in patients fit to undergo a major procedure and when a negative margin can be expected [5]. The achievement of R0 resection can be facilitated by venous resection as recent evidence shows that venous resection and reconstruction is feasible and safe, and with no significant increase in the mortality rate in dedicated centers [6–8].

Radiofrequency (RF) ablation is an accepted treatment to provide curative treatment for primary and secondary liver tumors, because of its safety profile, ease of use, minimal invasiveness and a high level of effectiveness [9, 10]. The use of intraoperative RF ablation in PDAC has also shown some interesting results in terms of safety and median survival [11, 12]. Bassi *et al.* demonstrated that open RF ablation of PDAC is feasible with an acceptable rate of complications [13]. A few studies on stage III unresectable locally advanced PDAC have shown that median survival after RF ablation ranged from 20 months to 33 months [14–16].

It is not uncommon during PD to find local invasion that renders the tumor unresectable even in cases where preoperative imaging didn’t show clear vascular infiltration. This can also happen at a late stage in the procedure when the pancreatic neck has been already divided, revealing vascular invasion of the superior mesenteric vessels. In these cases, when venous and/or arterial resection is not feasible or warranted, RF could be used as a palliative procedure that can provide possible advantages due to maximal tumor destruction and potential immune-stimulation.

Here we describe a surgical technique of PD utilizing an RF ablation device that assisted resection in a group of 6 patients with locally advanced PDAC with vascular involvement in whom venous resection was judged inappropriate. Moreover, RF could offer an advantage in survival as it has been shown that RF can activate the immune system by inducing tumor heat shock protein (HSP), cytokines and T cells which further increase its antitumor action [17–20].

## RESULTS

The patients had a median age of 73 years (range 65–79 years) and a body mass index (BMI) of 27.7 ± 4.2 kg/m^2^. Demographics and clinical characteristics of patients are shown in Table 1

**Table 1:** Demographics and clinical characteristics of patients in the study group

Parameters	Numbers
Total number of patients	6
Age (median), yrs. (range)	73 (65–79)
Male/Female ratio	3/3
Body max index (mean ± SD) Kg/m^2^	27.7 ± 4.2
Comorbidities:	
Type 2 Diabetes Mellitus	2
Essential hypertension	4
Chronic obstructive pulmonary disease	1
CA 19-9 (mean ± SD) IU	948.75 ± 674.34

Abbreviations: SD: standard deviation of the mean.

The mean operative time was 526.50 ± 73.96 minutes with minimal intraoperative blood loss (Table 2). The decision not to proceed to venous resection was due to age and co-morbidities of patients in four cases, involvement of jejunal branch draining into SMV in one case, and excessive length of vessel resection required for vein reconstruction in one case.

**Table 2:** Perioperative outcomes

Parameters	Numbers
Total number of patients	6
Operation time (mean ± SD) minutes	526.50 ± 73.96
Blood transfusion	Nil
Intensive care unit stay (median), hours	24 hours
Length of hospital stay (median), days	16.1 (12–21)
Complications:	
Superficial wound infection	2/6
Delayed gastric emptying	1/6
Intra-abdominal collections	1/6
General complications	2/6
30 days mortality	Nil

Abbreviations: SD: standard deviation of the mean.

**Figure 1:**
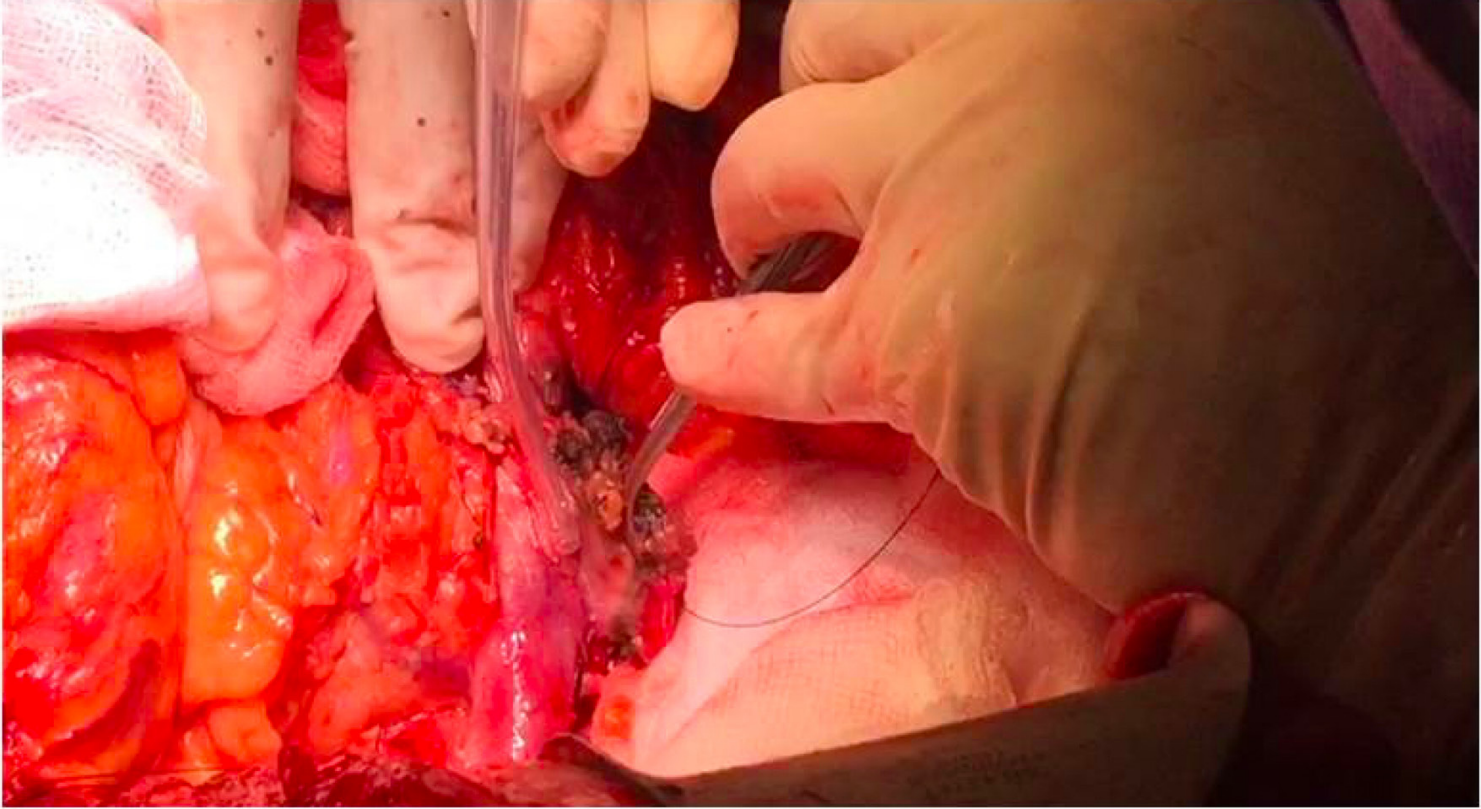
Ablated and desiccated tumor has been gently swept off the adherent vessel

In four patients, RF ablation caused tumor shrinkage and facilitated a plane of dissection, as there was no true vascular invasion. (Figure 1) Histopathology report of these cases showed an R1 resection, with moderately to poorly differentiated carcinoma T3 with nodal involvement in three cases.

In the other two cases, as there was tumor vascular invasion, the tumor could not be separated from the SMV. Part of the ablated tumor was then carefully resected off the vein with a scalpel staying close to the tumor vessel interface. The ablation of the tumor with temperatures reaching up to 90° C prior to resection eliminated the risk of tumor spillage. In these two cases, the ablation had a palliative intent and resulted in R2 resection, and the residual tumor on the vascular surface was left ablated. Histopathology report showed a poorly differentiated adenocarcinoma with nodal involvement (T4N1R2 in both cases).

There were no intra-operative complications and none of the patients received blood transfusion. All patients were admitted electively in intensive care unit with a stay of 24 hours. The median length of hospital stay was 16.1 days (range 12–21 days). Postoperative morbidity included two superficial wound infections and one central line infection; one patient had delayed gastric emptying; while one patient had postoperative psychosis, which did not prolong her hospital stay. There were no 30-day mortality, pancreatitis or deep abscess formation close to the ablated area. One patient was readmitted due to wound infection and small abdominal collections that were managed with antibiotic treatment without need for drainage (Table 2).

Of the four patients who had R1 resection, one patient is alive 22 months after the procedure with no evidence of recurrence; one is alive at 10 months; one alive at 5, and last one at 4 months, all with no evidence of recurrence. Of the two patients who had R2 resection, one is alive at 10 months with no evidence of recurrence and the other developed liver metastases two months after surgery, but he is currently alive at 16 months after PD. (Table 3) All patients had 3 months scan after surgery. All patients received or are receiving adjuvant chemotherapy.

**Table 3:** Survival data

Patient’s details	Alive at	Recurrence
1. 65 yrs., F; R1 - T3N0	22 months	No
2. 74 yrs., M; R2 - T4N1	16 months	Liver metastases
3. 79 yrs., M; R2 - T4N1	10 months	No
4. 76 yrs., F; R1 - T3N1	10 months	No
5. 71 yrs., F; R1 - T3N1	5 months	No
6. 74 yrs., M; R1 - T3N1	4 months	No

Abbreviations: F: female, M: male.

## DISCUSSION

Surgical resection is considered the only curative treatment for PDAC when a R0 resection is achieved. However, a significant number of patients are diagnosed at a locally advanced stage with vascular involvement, which limits curative resection (R0). The overall 5-year survival rate for locally advanced PDAC remains dismal [21]. In the presence of vascular involvement, R0 resections require resection and reconstruction of vascular structures, which is achieved in only a small group of patients [22–24]. As venous and arterial reconstruction increases the invasiveness of the procedure and could add to morbidity and possibly mortality, a risk and benefit analysis must be carefully considered for each patient. Since the beginning of the Whipple’s procedure era and its later establishment by Waugh and Clagett in 1946, clinicians have agreed that the main rule for surgery for malignant pancreatic tumors is that there should be a reasonable opportunity for cure, and the risk of death and morbidity should not outweigh the prospects for healing [25–27]. These principles act as a counterbalance between radical non-curative surgery and attempts to limit postoperative morbidity and mortality.

In some cases it is still difficult to differentiate intraoperatively between frank venous invasion from adherence due to inflammatory adhesions, as shown by studies on PD with venous resection where true venous involvement was present in around 60 to 70% of the specimens [28]. Moreover, the rates of R0 resection in PD with venous involvement show that despite an extensive procedure, R0 is not always achieved, with an average of up to 30 to 40% of cases where a non-curative resection is found on specimen despite the extensive resection [8, 29]. Moreover, tumor-related factors, such as tumor biology and aggressiveness, may lead to R1 resection despite excellent surgical technique [30].

Taking these facts into consideration, RF-assisted PD for tumors in close proximity to major vessels could be a extra surgical option in patients where vascular resection is not feasible or warranted.

In this current series the number of patients is very small (six patients) with very limited follow up (only 2 patients with follow up longer then 12 months), thus only preliminary results are presented. However, RF seemed to be safe in view of low morbidity and zero mortality and could represent an additional tool available to the pancreatic surgeon to locally destroy tumor that has been found to be unresectable at a late stage in the procedure.

The safety of RF in the treatment of unresectable PDAC has been demonstrated in palliative percutaneous or open procedures for PDAC where ablation was performed at the same time of palliative bypass [11–16, 31, 32]. Various studies have reported the use of RF devices to ablate (without resection) unresectable locally advanced PDAC with median survival benefit ranging from 20–33 months [11, 12]. As RF produces tumor shrinkage, RF ablation could achieve local control in larger PDAC or even tumor regression of smaller PDAC, as shown in a small series where RF ablation was applied to the unresectable pancreatic tumor when a palliative bypass was performed [31]. In our Institution as well as others, RF in pancreatic cancer has also been safely applied to the tumor via endoscopic ultrasound and endoscopically in case of malignant biliary obstruction before metal stent insertion and to unblock occluded stent with benefits in survival [33–37].

A potential advantage of RF is immunomodulation, which has been reported to have a positive role in improved outcomes following application of RF devices for various tumor types. Studies in both animals and *in vivo* human liver cells have reported increased expression levels of heath shock protein HSP-70, HSP-90, and glycoprotein 96, as well as translocation of nuclear high-mobility group protein B1 (HMGB1) expression in the periphery of the ablation zone i.e., transition zone. The enriched embodiment of HSP70 in tumor cells promotes the presentation of peptides on the tumor cell surface via an enhanced MHC class I expression and recognition by T cells and dendritic cells [38–41]. The post RF ablation induced immune changes in the periphery of the tumor have been considered as evidence of systemic immunomodulatory effects. In a study of hepatocellular carcinoma (HCC), Zerbini *et al.*, 2006 reported an increased percentage of activated T cells and circulating NK cells [42]. Similarly, the histopathology of the PD specimen following endoscopic RF ablation showed increased infiltration of inflammatory cells adjacent to the area of necrosis caused by the ablation [36]. This can yield a local vaccine effect that not only phagocytoses the debris and tumor cells, but also produces systemic immunomodulatory changes that may help in clearing up extra-pancreatic micro metastases, decreasing tumor recurrence and offering a potential increase in survival [18, 43–45].

Radiofrequency ablation has a direct effect by inducing necrosis of the tissue that is ablated. At the same time, different studies have demonstrated significant indirect effects in the areas around the ablated zone, where the cells become more sensitive to chemotherapy and radiation, and immodulation of the immune system involving pro-inflammatory cytokines, lymphocytes and antibodies, leading to acquired antitumor antigen-specific immunity [46]. As we have recently shown, outcomes of patients with hepatocellular carcinoma who had RF-assisted liver resections were better compared with other resection techniques and this could be due to the systemic and local immunomodulatory effect of RF [47]. While much of this evidence arises from studies conducted for organs other than the pancreas, Bassi *et al.* recently demonstrated that RF lead to early significant increase of IL-6 proinflammatory chemokine with lack of activation of immunosuppressive lymphocytes (CD4+CD25+Foxp3+ regulatory T cells), monocytes and plasmocytoid dendritic cells [48]. The latter provides scientific rationale for the reported improves survival after RF ablation in PDAC. Moreover, it has been recently observed that RF induced an adaptive response with a decrease in immunosuppression in ten patients undergoing open ablation for locally advanced PDAC [49].

In the same way that the therapeutic efficacy of RF ablation has become the gold standard for patients with small liver tumors, its use in locally advanced PDAC unsuitable for extensive resection could possibly offer a similar benefit. This report has considerable limitations as it only includes 6 patients with short follow up. However, it shows the feasibility and primary safety of this surgical procedure. In summary, this procedure could be considered as an additional technique in elderly patients with multiple associated comorbidities. The potential effect of RF ablation on the immune system may also offer an added effect when used in advanced PDAC, especially with potential combination with check-point inhibitors that may further strengthen its role in treating this dismal disease. The authors believe that conventional PD with vascular reconstruction should be attempted and is the procedure of choice. However, if radical curative surgery is not possible, then RF assisted pancreatic resection could be an option to selected patients for palliation of locally advanced tumor.

## MATERIALS AND METHODS

### Study design

We report a series of 6 patients who underwent a RF-assisted PD at our Hepatobiliary and Pancreatic Centre between December 2015 and June 2017 for a locally advanced PDAC. All cases were discussed in a multidisciplinary team meeting and surgery with curative intent was recommended on the basis of preoperative investigations. However, intraoperatively the tumor was not resectable without vascular resection/reconstruction that was considered not safe in these 6 cases. Thus, RF was instead applied to the tumor with palliative intent. Parameters examined included age, gender, co-morbidities, and characteristics of the tumor. The primary outcome variables were 30-day postoperative morbidity and overall 30-day postoperative mortality. Secondary outcome variables included operative time, need for blood transfusion, specific postoperative complications (including bleeding, wound infection, collections, sepsis, anastomotic complications, and general complications), need for reoperation within 30 days, and intensive care re-admission and postoperative length of stay. Follow up data were collected from the date of surgery till October 2017. All data were entered into a Microsoft Excel^™^ database for statistical analysis. The data was analyzed by using MedCalc_2004 software (ver. 7.4.3.0).

### Surgical procedure

**Figure 2:**
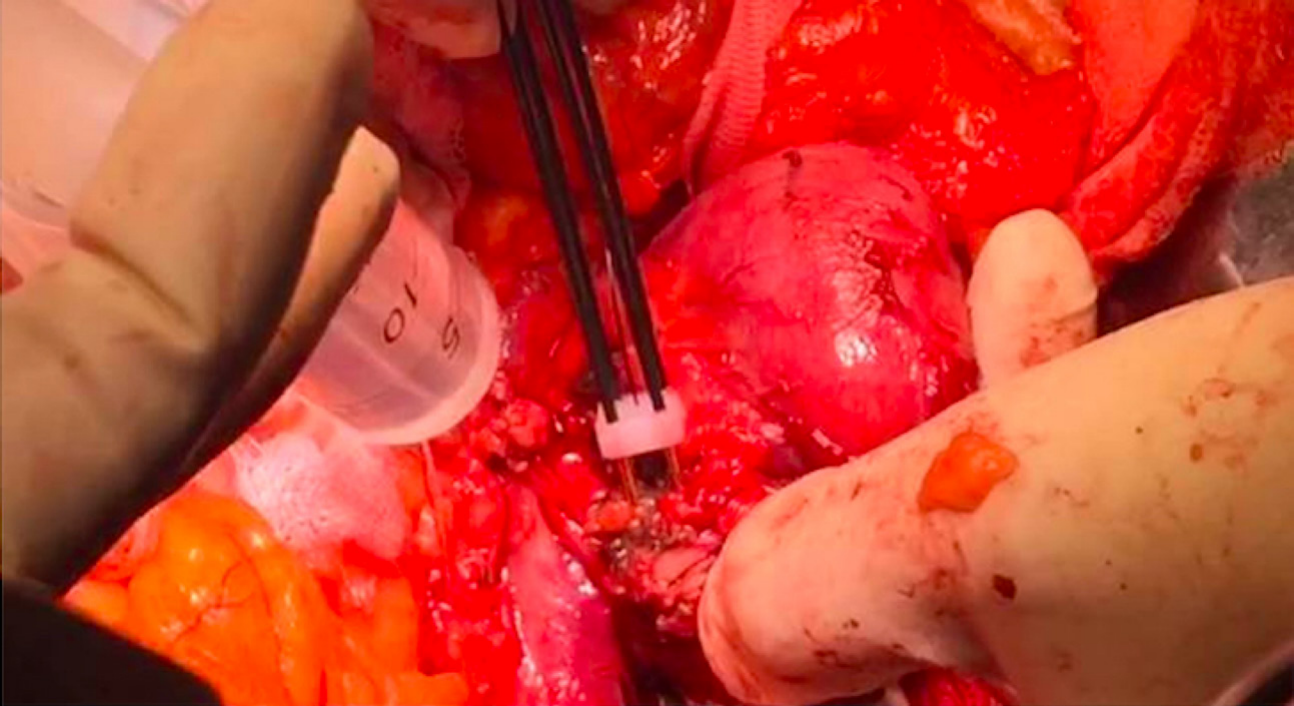
Sequential application of RF probes to create parallel ablation lines adjacent to the tumor vessel interface

**Figure 3:**
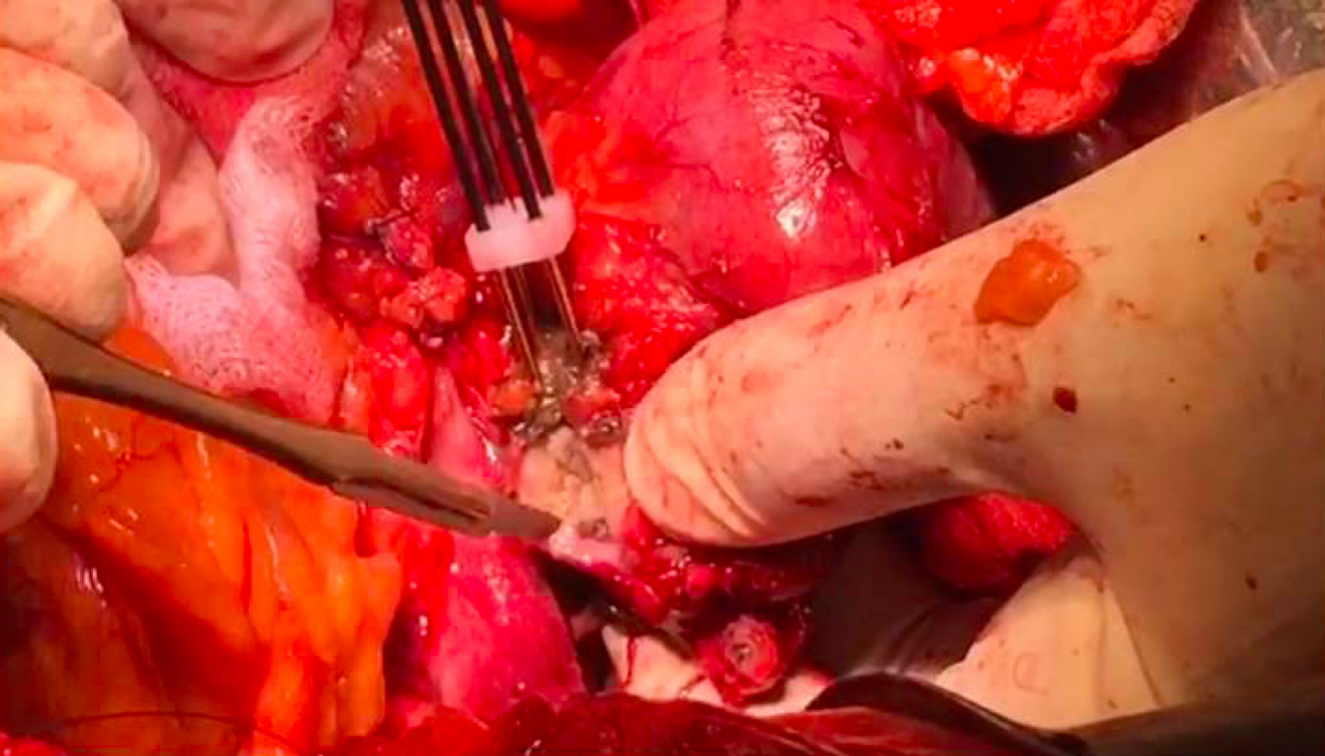
Resection with scalpel over the ablated region of tumor at tumor vessel interface

The surgical approach to PDAC of the pancreatic head was similar to a conventional PD except that after division of the neck of the pancreas, the Habib^™^ 4X (AngioDynamics Inc., USA) was used to ablate the tumor. A parallel line of ablation was created by sequential applications of RF-energy at 60 watts. This resulted in coagulative necrosis of the tumor tissue. The first vertical line of ablation was performed approximately 5–10 mm away from the involved vessel, and then a second parallel line of ablation was made further away to it (Figure 2). Subsequently, several transverse applications were required to create a third line of ablation, which connected with the vertical parallel lines to ensure complete ablation. Cold water was poured continuously over the mesenteric vessels during the ablation process to avoid thermal injury. The probe was moved swiftly in a seesaw fashion over 3–5 mm at its axis of application in order to avoid any tumor tissue adherence. This helped to create a 1 cm ablated and coagulated margin of tissue which could be cut across (Figure 3). Each time the ablation lasted less than 15 minutes.

## References

[1] Siegel RL, Miller KD, Jemal A (2016). Cancer statistics, 2016. CA Cancer J Clin.

[2] Christians K, Evans DB (2009). Pancreaticoduodenectomy and vascular resection: persistent controversy and current recommendations. Ann Surg Oncol.

[3] Howard TJ, Krug JE, Yu J, Zyromski NJ, Schmidt CM, Jacobson LE, Madura JA, Wiebke EA, Lillemoe KD (2006). A margin-negative R0 resection accomplished with minimal postoperative complications is the surgeon's contribution to long-term survival in pancreatic cancer. J Gastrointest Surg.

[4] Sohn TA, Yeo CJ, Cameron JL, Koniaris L, Kaushal S, Abrams RA, Sauter PK, Coleman J, Hruban RH, Lillemoe KD (2000). Resected adenocarcinoma of the pancreas-616 patients: results, outcomes, and prognostic indicators. J Gastrointest Surg.

[5] NCCN (2017). National Comprehensive Cancer Network, Clinical Practice Guidelines in Oncology: Pancreatic Adenocarcinoma (Version 2.2017).

[6] Gong Y, Zhang L, He T, Ding J, Zhang H, Chen G, Zhang D, Wu Z, Chen Q, Fan H, Wang Q, Bie P, Wang H (2013). Pancreaticoduodenectomy combined with vascular resection and reconstruction for patients with locally advanced pancreatic cancer: a multicenter, retrospective analysis. PLoS One.

[7] Cheung TT, Poon RT, Chok KS, Chan AC, Tsang SH, Dai WC, Chan SC, Fan ST, Lo CM (2014). Pancreaticoduodenectomy with vascular reconstruction for adenocarcinoma of the pancreas with borderline resectability. World J Gastroenterol.

[8] Yu XZ, Li J, Fu DL, Di Y, Yang F, Hao SJ, Jin C (2014). Benefit from synchronous portal-superior mesenteric vein resection during pancreaticoduodenectomy for cancer: a meta-analysis. Eur J Surg Oncol.

[9] McDermott S, Gervais DA (2013). Radiofrequency ablation of liver tumors. Semin Intervent Radiol.

[10] Curley SA (2003). Radiofrequency ablation of malignant liver tumors. Ann Surg Oncol.

[11] Fegrachi S, Besselink MG, van Santvoort HC, van Hillegersberg R, Molenaar IQ (2014). Radiofrequency ablation for unresectable locally advanced pancreatic cancer: a systematic review. HPB.

[12] Pandya GJ, Shelat VG (2015). Radiofrequency ablation of pancreatic ductal adenocarcinoma: The past, the present and the future. World J Gastrointest Oncol.

[13] Girelli R, Frigerio I, Salvia R, Barbi E, Tinazzi Martini P, Bassi C (2010). Feasibility and safety of radiofrequency ablation for locally advanced pancreatic cancer. Br J Surg.

[14] Girelli R, Giardino A, Frigerio I, Salvia R, Partelli S, Bassi C (2011). Survival after radiofrequency of stage III pancreatic carcinoma: a wind of change?. HPB.

[15] Spiliotis JD, Datsis AC, Michalopoulos NV, Kekelos SP, Vaxevanidou A, Rogdakis AG, Christopoulou AN (2007). Radiofrequency ablation combined with palliative surgery may prolong survival of patients with advanced cancer of the pancreas. Langenbecks Arch Surg.

[16] Matsui Y, Nakagawa A, Kamiyama Y, Yamamoto K, Kubo N, Nakase Y (2000). Selective thermocoagulation of unresectable pancreatic cancers by using radiofrequency capacitive heating. Pancreas.

[17] Hiroishi K, Eguchi J, Baba T, Shimazaki T, Ishii S, Hiraide A, Sakak i M, Doi H, Uozumi S, Omori R, Matsumura T, Yanagawa T, Ito T, Imawari M (2010). Strong CD8(+) T-cell responses against tumor-associated antigens prolong the recurrence-free interval after tumor treatment in patients with hepatocellular carcinoma. J Gastroenterol.

[18] Waitz R, Solomon SB (2009). Can local radiofrequency ablation of tumors generate systemic immunity against metastatic disease?. Radiology.

[19] Mazzaglia PJ, Berber E, Siperstein AE (2007). Radiofrequency thermal ablation of metastatic neuroendocrine tumors in the liver. Curr Treat Options Oncol.

[20] Keisari Y, Hochman I, Confino H, Korenstein R, Kelson I (2014). Activation of local and systemic anti-tumor immune responses by ablation of solid tumors with intratumoral electrochemical or alpha radiation treatments. Cancer Immunol Immunother.

[21] Miller KD, Siegel RL, Lin CC, Mariotto AB, Kramer JL, Rowland JH, Stein KD, Alteri R, Jemal A (2016). Cancer treatment and survivorship statistics, 2016. CA Cancer J Clin.

[22] Verbeke CS (2013). Resection margins in pancreatic cancer. Surg Clin North Am.

[23] Sugiura T, Uesaka K, Mihara K, Sasaki K, Kanemoto H, Mizuno T, Okamura Y (2013). Margin status, recurrence pattern, and prognosis after resection of pancreatic cancer. Surgery.

[24] Carroll JE, Smith JK, Simons JP, Murphy MM, Ng SC, Shah SA, Zhou Z, Tseng JF (2010). Redefining mortality after pancreatic cancer resection. J Gastrointest Surg.

[25] Waugh JM, Clagett OT (1946). Resection of the duodenum and head of the pancreas for carcinoma; an analysis of thirty cases. Surgery.

[26] Hirano S, Kondo S, Hara T, Ambo Y, Tanaka E, Shichinohe T, Suzuki O, Hazama K (2007). Distal pancreatectomy with en bloc celiac axis resection for locally advanced pancreatic body cancer: long-term results. Ann Surg.

[27] Ahn YJ, Kim SW, Park YC, Jang JY, Yoon YS, Park YH (2003). Duodenal-preserving resection of the head of the pancreas and pancreatic head resection with second-portion duodenectomy for benign lesions, low-grade malignancies, and early carcinoma involving the periampullary region. Arch Surg.

[28] Tang D, Zhang JQ, Wang DR (2011). Long term results of pancreatectomy with portal-superior mesenteric vein resection for pancreatic carcinoma: a systematic review. Hepatogastroenterology.

[29] Giovinazzo F, Turri G, Katz MH, Heaton N, Ahmed I (2016). Meta-analysis of benefits of portal-superior mesenteric vein resection in pancreatic resection for ductal adenocarcinoma. Br J Surg.

[30] Wang F, Gill AJ, Neale M, Puttaswamy V, Gananadha S, Pavlakis N, Clarke S, Hugh TJ, Samra JS (2014). Adverse tumor biology associated with mesenterico-portal vein resection influences survival in patients with pancreatic ductal adenocarcinoma. Ann Surg Oncol.

[31] Hadjicostas P, Malakounides N, Varianos C, Kitiris E, Lerni F, Symeonides P (2006). Radiofrequency ablation in pancreatic cancer. HPB.

[32] Varshney S, Sewkani A, Sharma S, Kapoor S, Naik S, Sharma A, Patel K (2006). Radiofrequency ablation of unresectable pancreatic carcinoma: feasibility, efficacy and safety. JOP.

[33] Kallis Y, Phillips N, Steel A, Kaltsidis H, Vlavianos P, Habib N, Westaby D (2015). Analysis of Endoscopic Radiofrequency Ablation of Biliary Malignant Strictures in Pancreatic Cancer Suggests Potential Survival Benefit. Dig Dis Sci.

[34] Brauer BC (2015). Intraductal Radiofrequency Ablation (RFA) for Pancreatic Cancer: Getting in Under the Wire?. Dig Dis Sci.

[35] Figueroa-Barojas P, Bakhru MR, Habib NA, Ellen K, Millman J, Jamal-Kabani A, Gaidhane M, Kahaleh M (2013). Safety and efficacy of radiofrequency ablation in the management of unresectable bile duct and pancreatic cancer: a novel palliation technique. J Oncol.

[36] Law R, Pai M, Baron T, Habib N (2013). The effects of endobiliary radiofrequency ablation in two patients with pancreatic cancer: Gross and microscopic findings. Gastrointestinal Intervention.

[37] Song TJ, Seo DW, Lakhtakia S, Reddy N, Oh DW, Park DH, Lee SS, Lee SK, Kim MH (2016). Initial experience of EUS-guided radiofrequency ablation of unresectable pancreatic cancer. Gastrointest Endosc.

[38] Jarnicki AG, Lysaght J, Todryk S, Mills KH (2006). Suppression of antitumor immunity by IL-10 and TGF-beta-producing T cells infiltrating the growing tumor: influence of tumor environment on the induction of CD4+ and CD8+ regulatory T cells. J Immunol.

[39] Yu P, Lee Y, Liu W, Krausz T, Chong A, Schreiber H, Fu YX (2005). Intratumor depletion of CD4+ cells unmasks tumor immunogenicity leading to the rejection of late-stage tumors. J Exp Med.

[40] Todryk SM, Melcher AA, Dalgleish AG, Vile RG (2000). Heat shock proteins refine the danger theory. Immunology.

[41] Osterloh A, Breloer M (2008). Heat shock proteins: linking danger and pathogen recognition. Med Microbiol Immunol (Berl).

[42] Zerbini A, Pilli M, Ferrari C, Missale G (2006). Is there a role for immunotherapy in hepatocellular carcinoma?. Dig Liver Dis.

[43] Gameiro SR, Higgins JP, Dreher MR, Woods DL, Reddy G, Wood BJ, Guha C, Hodge JW (2013). Combination therapy with local radiofrequency ablation and systemic vaccine enhances antitumor immunity and mediates local and distal tumor regression. PLoS One.

[44] Rozenblum N, Zeira E, Bulvik B, Gourevitch S, Yotvat H, Galun E, Goldberg SN (2015). Radiofrequency Ablation: Inflammatory Changes in the Periablative Zone Can Induce Global Organ Effects, including Liver Regeneration. Radiology.

[45] Kim YS, Rhim H, Lim HK, Choi D, Lee MW, Park MJ (2011). Coagulation necrosis induced by radiofrequency ablation in the liver: histopathologic and radiologic review of usual to extremely rare changes. Radiographics.

[46] Paiella S, Salvia R, Ramera M, Girelli R, Frigerio I, Giardino A, Allegrini V, Bassi C (2016). Local Ablative Strategies for Ductal Pancreatic Cancer (Radiofrequency Ablation, Irreversible Electroporation): A Review. Gastroenterol Res Pract.

[47] Huang KW, Lee PH, Kusano T, Reccia I, Jayant K, Habib N (2017). Impact of cavitron ultrasonic surgical aspirator (CUSA) and bipolar radiofrequency device (Habib-4X) based hepatectomy for hepatocellular carcinoma on tumour recurrence and disease-free survival. Oncotarget.

[48] Giardino A, Innamorati G, Girelli R, Frigerio I, Regi P, Scopelliti F, Paiella S, Pederzoli P, Bassi C, Butturini G (2017). Radiofrequency ablation of Locally Advanced Pancreatic Cancer: Immunostimulation Patterns. 12th Biennal E-AHPBA Congress. Mainz.

[49] Giardino A, Innamorati G, Ugel S, Perbellini O, Girelli R, Frigerio I, Regi P, Scopelliti F, Butturini G, Paiella S, Bacchi on M, Bassi C, Pederzoli P (2017). Immunomodulation after radiofrequency ablation of locally advanced pancreatic cancer by monitoring the immune response in 10 patients. Pancreatology.

